# Comprehensive comparison of three different workstations for accurate planning of endovascular stent implantation in patients with thoracic aortic aneurysms

**DOI:** 10.1016/j.ejro.2022.100427

**Published:** 2022-06-16

**Authors:** Vitali Koch, Gerald Loos, Leon D. Gruenewald, Katrin Eichler, Christian Booz, Tommaso D’Angelo, Ibrahim Yel, Scherwin Mahmoudi, Simon S. Martin, Marc Harth, Moritz H. Albrecht, Stephan Zangos, Simon Bernatz, Axel Thalhammer, Jan-Erik Scholtz, Thomas J. Vogl, Tatjana Gruber-Rouh

**Affiliations:** a Department of Diagnostic and Interventional Radiology, University Hospital Frankfurt, Frankfurt am Main, Germany; b Department of Biomedical Sciences and Morphological and Functional Imaging, University Hospital Messina, Messina, Italy

**Keywords:** CTA, Computed tomography angiography, PACS, Picture archiving and communication system, PVC, Polyvinyl chloride, TAA, Thoracic aortic aneurysm, TEVAR, Thoracic endovascular aortic repair, Thoracic aortic aneurysm, Endovascular aortic repair, Workstation comparison, Diagnostic accuracy, Computed tomography

## Abstract

**Purpose:**

To assess the diagnostic precision of three different workstations for measuring thoracic aortic aneurysms (TAAs) in vivo and ex vivo using either pre-interventional computed tomography angiography scans (CTA) or a specifically designed phantom model.

**Methods:**

This retrospective study included 23 patients with confirmed TAA on routinely performed CTAs. In addition to phantom tube diameters, one experienced blinded radiologist evaluated the dimensions of TAAs on three different workstations in two separate rounds. Precision was assessed by calculating measurement errors. In addition, correlation analysis was performed using Pearson correlation.

**Results:**

Measurements acquired at the Siemens workstation deviated by 3.54% (range, 2.78–4.03%; *p* = 0.14) from the true size, those at General Electric by 4.05% (range, 1.46–7.09%; *p* < 0.0001), and at TeraRecon by 4.86% (range, 3.22–6.45%; *p* < 0.0001). Accordingly, Siemens provided the most precise workstation at simultaneously most fluctuating values (scattering of 4.46%). TeraRecon had the smallest fluctuation (scattering of 2.83%), but the largest deviation from the true size of the phantom. The workstation from General Electric showed a scattering of 2.94%. The highest overall correlation between the 1st and 2nd rounds was observed with measurements from Siemens (r = 0.898), followed by TeraRecon (r = 0.799), and General Electric (r = 0.703). Repetition of measurements reduced processing times by 40% when using General Electric, by 20% with Siemens, and by 18% with TeraRecon.

**Conclusions:**

In conclusion, all three workstations facilitated precise assessment of dimensions in the majority of cases at simultaneously high reproducibility, ensuring accurate pre-interventional planning of thoracic endovascular aortic repair.

## Highlights

• Pre-interventional planning of TEVAR in patients with TAAs using CTA is feasible.
• All three workstations facilitated accurate measurements in vivo and ex vivo.
• Repetition of measurements resulted in faster processing due to learning effects.

## 1 Introduction

Thoracic aortic aneurysms (TAAs) represent a potentially life-threatening disease that requires immediate detection to prevent complications arising from a delayed diagnosis, such as aortic dissection or rupture [1]. In an aging population with continuously increasing cardiovascular risk factors, the annual incidence of currently 3 cases per 100.000 per year is likely to keep rising [2].

Management strategies include surgical and endovascular techniques, depending on many factors including size, location, growth, and associated comorbidities of the individual patient [3], [4], [5]. Considering the annual risk for aortic rupture or dissection of up to 7% for TAAs > 60 mm, current guidelines recommend surgery at sizes of ≥ 55 mm in most cases [6], [7], [8]. In the case of genetic disorders or bicuspid aortic valve, intervention is typically recommended at lower values [9].

Along with improvements in availability and material, endovascular techniques attracted scientific attention aiming at restoring cardiovascular circulation through the implantation of a membrane-covered stent graft [9], [10]. In this context, careful pre-interventional planning and anatomical visualization are crucial to ensure a successful thoracic endovascular aortic repair (TEVAR) [11]. Contrast-enhanced computed tomography plays a central role in the assessment and characterization of aneurysms by providing three-dimensional information about adjacent structures and vasculature. It is widely available and represents an optimal preoperative imaging modality given its ability to measure aneurysmal morphology accurately and precisely [6].

For pre-procedural planning of endovascular stent insertion, different CT workstations have been developed that provide miscellaneous options to visualize aortic lesions in three-dimensional models and evaluate both the diameter and length of the healthy proximal and distal landing zones [12]. To date, data about the diagnostic accuracy, reliability, and reproducibility of CT-based measurements using workstations from different manufacturers are sparse. Therefore, the purpose of the present study was to evaluate the diagnostic precision of three different workstations in assessing TAA dimensions using either computed tomography angiography scans (CTA) of patients or a specifically designed phantom model.

## 2 Methods

The institutional ethical review board approved this retrospective study that complies with the Declaration of Helsinki. The need for written informed consent was waived.

### 2.1 Study population

The study population consisted of 23 patients with complete CT data sets and sufficient image quality. Additionally, a phantom was constructed to compare measurements with fixed true values.

### 2.2 CT scan protocol

Contrast-enhanced CT scans were carried out on a third-generation dual-source dual-energy CT scanner (Somatom Force; Siemens Healthineers, Forchheim, Germany). The system consisted of two beams operating at a lower and higher tube voltage (tube A, 90 kVp and 180 mAs; tube B, Sn150 kVp [0.64 mm tin filter] and 180 mAs) using automatic attenuation-based tube current modulation (CARE Dose 4D; Siemens Healthineers, Forchheim, Germany). All CT scans were performed in the craniocaudal scan direction. Images were acquired at three different phases, an unenhanced as well as a contrast-enhanced venous and arterial phase. Scans were ECG-gated and performed using a conventional protocol with the following parameters: 120 kV, 70 mAs, 0.6 mm slice thickness, and 1 mm collimation. According to standard protocols in clinical routine, patients received varying doses of a non-ionic monomeric contrast agent (Bracco Imaging Deutschland GmbH, Konstanz, Germany) at a flow rate of 2–4 mL/s adjusted to the individual bodyweight of patients. For automated bolus tracking, a region of interest was placed in the ascending aorta with a threshold of 120 Hounsfield units (HU) to time the start of the arterial phase. The venous phase was performed with a delay of 80-90 s after injection start. All data sets were sent to the picture archiving and communication system (PACS Centricity, Version 4.2; General Electric Healthcare, Solingen, Germany) for further postprocessing.

### 2.3 Specifications of the three different workstations

In this study, three different workstations for the postprocessing of CT images were examined: Aquarius (Version 3.7.0.12; TeraRecon, Durham, USA), Syngo (Version VE32B; Siemens Healthineers, Forchheim, Germany), and Volume Share 2 (Version AW.4.4; General Electric Healthcare, Waukesha, USA).

All workstations were purchased with dedicated integrated vendor-specific software that was directly connected to the PACS allowing for three-dimensional visualization of the aorta. A detailed description of all three workstations is available in Supplemental material.

### 2.4 Assessment of patient data sets

All CTA series were analyzed twice by one single radiologist (J.E.S., board-certified radiologist with 9 years of experience in CT imaging) in a randomized blinded fashion on different days. The radiologist was familiar with the handling of workstations but had no previous experience with the three systems investigated in the present study. For randomization, both patients and workstations have been assigned to a distinct number, written on cards, and shuffled. Finally, each patient was randomly allocated to one of the three workstations. One week after the complete evaluation of all 23 patients on each workstation, a 2nd round has been initiated to assess the repeatability and correlation of measurements with the 1st round. Patients were not randomized again and evaluated in the same order as in the 1st round. This evaluation aimed to answer the question of whether a learning effect resulted in faster processing of patient data sets. Assessment time for the analysis of each data set was noted.

In detail, the following measurements were made:

1. Diameter of the aorta after branching of the left subclavian artery.
2. Diameter of the aorta before the beginning of the aortic lesion.
3. Greatest diameter of the vessel lumen within the aortic lesion.
4. Greatest diameter of the thrombus within the aortic lesion.
5. Diameter of the aorta immediately after the end of the aortic lesion.
6. Diameter of the aorta before the origin of the coeliac trunk.
7. Diameter of the common femoral arteries on both sides shortly after their origin.
8. Distance between the origin of the left subclavian artery and the beginning of the aortic lesion.
9. Length of the aortic lesion.
10. Distance between the end of the aortic lesion and the coeliac trunk.
11. Total distance between the origin of the left subclavian artery and the coeliac trunk.

### 2.5 Phantom

**Fig. 1:**
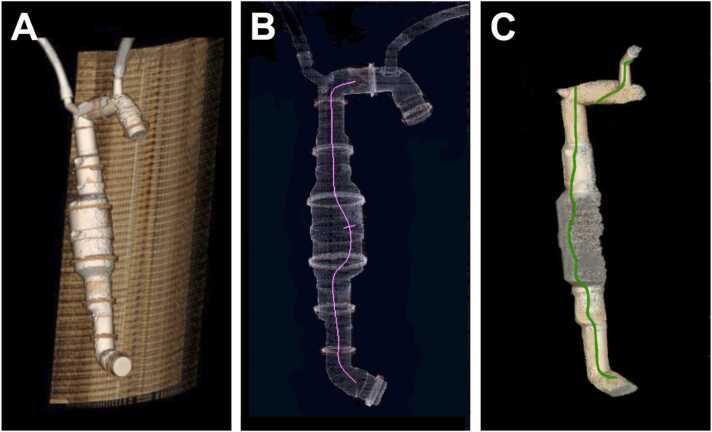
Three-dimensional illustration of the phantom using A) TeraRecon, B) Siemens after inserting the central axis (shown as purple line), and C) General Electric with the depiction of the central axis (shown as green line) prior to manual adjustments. The greyish zone in the middle of the phantom (C) is notable which corresponds to numerous air bubbles collected at the aneurysmatic phantom lesion.

In addition to CTAs from 23 patients, a phantom was constructed using commercially available polyvinyl chloride (PVC) tubes with known dimensions of the individual components (Fig. 1). The phantom represented a simplified aortic model revealing the most important anatomical corner points. In this context, the origins of the brachiocephalic trunk and the left subclavian artery were particularly important as the starting points for several measurements. Phantoms of similar construction have been proven extremely useful in previous studies to simulate all procedural steps of an endovascular aortic repair [13].

**Fig. 2:**
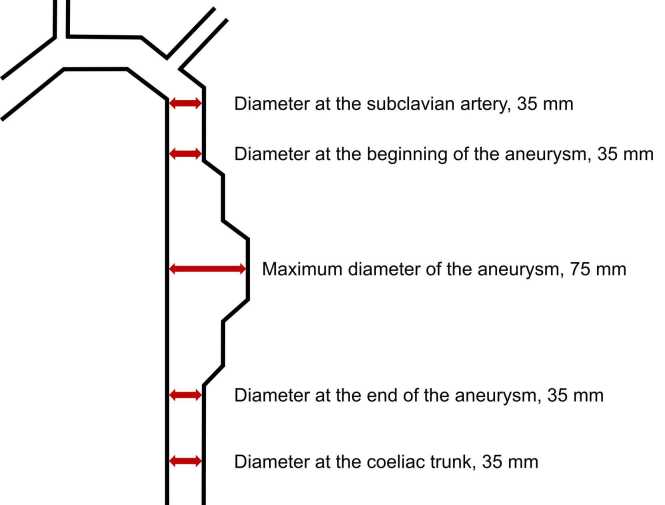
Schematic illustration of the phantom showing its true diameters at different predefined measurement points.

The closed tube system was filled with contrast medium and water at a ratio of 1:10 and coated with a significant number of air bubbles on the inside, imitating arteriosclerotic plaques of a natural aorta. The inner diameters could be measured with a caliper to within a tenth of a millimeter and served as reference values for the semi-automated measurements at the respective workstation. To keep the discrepancy between manually measured and fixed diameters as small as possible, positions without air bubbles on the edge of the tubes were preferably chosen. Phantom tube diameters had the following predefined and strictly standardized true values (Fig. 2):

**Table:** 

**Measurement points**	**True value (mm)**
Tube diameter at the artificial subclavian artery	35
Tube diameter at the beginning of the aneurysm	35
Maximum tube diameter of the aneurysm	75
Tube diameter at the end of the aneurysm	35
Tube diameter at the coeliac trunk	35
Distance between the aneurysm and the artificial subclavian artery	50
Length of the aneurysm	300
Distance between the aneurysm and the artificial coeliac trunk	50
Total distance between the origin of the artificial subclavian artery and the coeliac trunk	400

**Fig. 3:**
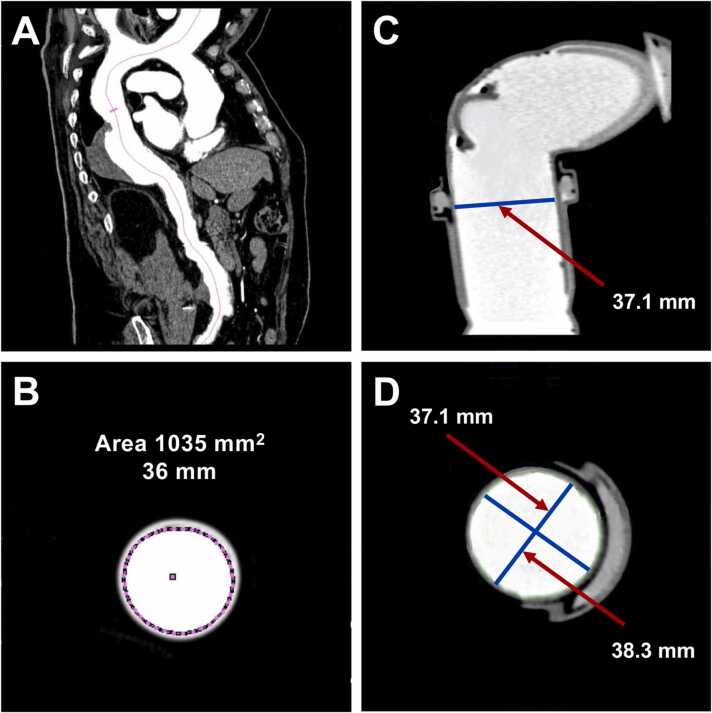
Illustration of measurements acquired at the Siemens workstation on coronal and axial images. The purple line represents the central axis (A) and the inner diameter of the aorta (B) in CTA scans. Measurements of phantom tube diameters are shown in coronal (C) and axial views (D). The blue lines represent the inner tube diameter.

To compare against those fixed reference values, each defined point was measured ten times at the three workstations, respectively. The respective ten-part series of measurements from each point and each workstation was compared with the known dimensions of the phantom. Spiral CT data sets of the phantom were processed exactly like that of a patient. Fig. 3 illustrates the positioning of measurements in CTAs and phantom tubes.

### 2.6 Statistical analysis

Statistical analysis was conducted with MedCalc for Windows (Version 18; MedCalc, Ostend, Belgium). The normal distribution of datasets was tested using the Shapiro-Wilk test. Accordingly, results are expressed as mean ± standard deviation or median with interquartile ranges. A *p* value < 0.05 was considered statistically significant.

Comparisons between continuous variables were performed using one-way ANOVA, chi-square statistic tests, or two-tailed Student’s *t*-test, where appropriate. Correlation analysis between patient data sets acquired in the two rounds, between workstations, and between measured and true phantom dimensions was performed by calculating Pearson product-moment correlation (r) and linear regression. An r value of less than 0.40, 0.41–0.60, 0.61–0.80, and greater than 0.80 was considered as poor, moderate, strong, and very strong, respectively. Measurement errors were defined as the difference between measured and true dimensions of the phantom:

Measurementerror(mm)=Measureddiameter⁢minustruediameter

$$\mathrm{Relative}\;\mathrm{measurement}\;error\;(\%)=\frac{\mathrm{Measurement}\;error}{\mathrm{True}\;diameter}\times 100$$

## 3 Results

A total of 23 patients (58 ± 10 years; range, 42–83), comprising 7 women and 16 men, were included in this study. Overall mean body mass index was 27 ± 3 kg/m^2^ (range, 23–32 kg/m^2^).

Aortic lesions could be classified into 15 dissections, 7 aneurysms, and 1 aortic rupture. Regarding aortic dissection, 15 aortic dissections could be allocated to Stanford B or Type 3 according to the DeBakey classification. The single rupture was located in aortic segment III. Patient characteristics are summarized in Table 1.

**Table 1:** Baseline characteristics of the study cohort.

**Characteristics of the study cohort**	**Value**
Number of overall patients (women; men)	23 (7; 16)
Overall mean age (y) ± SD, range	58 ± 10, 42–83
Overall mean BMI (kg/m²) ± SD, range	27 ± 3, 23–32
Mean age of women (y) ± SD, range (Mean BMI of women (kg/m²) ± SD, range)	57 ± 9, 46–70 (27 ± 2, 25–30)
Mean age of men (y) ± SD, range (Mean BMI of men (kg/m²) ± SD, range)	58 ± 11, 42–83 (28 ± 3, 23–32)

Abbreviations: BMI, body mass index.

### 3.1 Phantom

First, the phantom was scanned ten times at each workstation (Table 2) to compare measured values with the true dimensions of phantom tubes.

**Table 2:** Diagnostic accuracy of the phantom at different predefined and fixed measurement points.

	**True value (mm)**	**Average (mm)**	**95% confidence interval (CI)**	***p* value**
**Siemens Healthineers Syngo (Version VE32B)**				
Tube diameter at the subclavian artery	35	35.50	34.89–36.11	0.0957
Tube diameter at the beginning of the aneursym	35	36.40	36.03–36.77	< 0.0001
Maximum tube diameter of the aneurysm	75	74.90	74.67–75.13	0.3434
Tube diameter at the end of the aneurysm	35	36.30	35.95–36.65	< 0.0001
Tube diameter at the coeliac trunk	35	36.00	36.00–36.00	< 0.0001
Distance of the aneurysm to the subclavian artery	50	51.42	49.55–53.29	0.1194
Length of the aneurysm	300	290.00	281.15–298.85	0.0309
Distance of the aneurysm to the coeliac trunk	50	52.10	50.95–53.52	0.0085
Total distance between the origin of the subclavian artery and the coeliac trunk	400	393.52	384.38–402.66	0.1431

**TeraRecon Aquarius (Version 3.7.0.12)**				
Tube diameter at the subclavian artery	35	36.23	35.39–37.06	0.0088
Tube diameter at the beginning of the aneursym	35	37.07	36.45–37.68	< 0.0001
Maximum tube diameter of the aneurysm	75	76.44	74.91–77.97	0.0953
Tube diameter at the end of the aneurysm	35	37.28	36.82–37.75	0.0726
Tube diameter at the coeliac trunk	35	36.17	35.78–36.64	0.0004
Distance of the aneurysm to the subclavian artery	50	50.06	48.67–51.45	0.9268
Length of the aneurysm	300	281.81	278.75–284.88	< 0.0001
Distance of the aneurysm to the coeliac trunk	50	51.21	48.68–53.74	0.3078
Total distance between the origin of the subclavian artery and the coeliac trunk	400	383.08	379.17–386.98	< 0.0001

**General Electric Volume Share 2 (Version AW.4.4)**				
Tube diameter at the subclavian artery	35	35.73	35.43–36.03	0.0004
Tube diameter at the beginning of the aneursym	35	37.67	37.20–38.14	< 0.0001
Maximum tube diameter of the aneurysm	75	75.10	74.98–75.22	0.0957
Tube diameter at the end of the aneurysm	35	36.55	36.27–36.83	< 0.0001
Tube diameter at the coeliac trunk	35	37.27	37.11–37.44	< 0.0001
Distance of the aneurysm to the subclavian artery	50	48.84	46.57–51.10	0.2749
Length of the aneurysm	300	295.67	291.72–299.62	0.0349
Distance of the aneurysm to the coeliac trunk	50	42.44	40.83–44.75	< 0.0001
Total distance between the origin of the subclavian artery and the coeliac trunk	400	386.95	383.09–390.32	< 0.0001

Measurements of phantom tubes with a fixed diameter of 35 mm deviated generally from the true size, being the smallest (range of mean deviation, 0.5–1.4 mm) at the Siemens workstation, followed by General Electric (range, 0.7–2.7 mm) and TeraRecon (range, 1.2–2.3 mm).

Regarding diameters of 75 mm, both Siemens and General Electric showed results that are significantly better in terms of precision than the measurements of 35 mm tubes (mean deviation of 0.1 mm, respectively). The TeraRecon, on the other hand, differed 1.4 mm on average from the true size.

Scattering of measurements at 50 mm diameters can be considered similar across all three workstations (range, −1.2 to +2.1 mm). Only at the measurement point “distance of the aneurysm to the artificial coeliac trunk” General Electric was far below the true value (mean deviation of −7.6 mm) due to a user error.

At diameters of 300 mm, all three workstations felt below the true size of the phantom tube. The mean distance to the true value was 10 mm for Siemens, 8.2 mm for TeraRecon, and 4.3 mm for General Electric.

To summarize, measurements acquired at the Siemens workstation deviated by 3.54% on average (range, 2.78–4.03%; *p* = 0.14) from the true dimensions, those at General Electric by 4.05% (range, 1.46–7.09%; *p* < 0.0001), and at TeraRecon by 4.86% (range, 3.22–6.45%; *p* < 0.0001). Accordingly, Siemens was the most precise workstation at simultaneously most fluctuating values (scattering of 4.46%). TeraRecon had the smallest fluctuation (scattering of 2.83%), but the largest deviation from the true size of the phantom. The workstation from General Electric showed a scattering of 2.94%.

### 3.2 Patient data sets

Correlation coefficients between the 1st and 2nd round, both performed at one of the three different workstations, are shown in Table 3. The highest overall correlation between the 1st and 2nd round was observed with measurements from Siemens (r = 0.898, range 0.404–0.990), followed by TeraRecon (r = 0.799, range 0.314–0.992), and General Electric (r = 0.703, range 0.395–0.925). It is noticeable that the p values of most correlations were below 0.05 pointing towards a good reproducibility of measurements. Correlation coefficients with p values above 0.05 were found at the measurement points "diameter of the thrombus", "diameter of the right and left femoral artery", and "distance of the aneurysm to the subclavian artery".

**Table 3:** Correlation analysis between the 1st and 2nd round at every single workstation.

	**General Electric**		**TeraRecon**		**Siemens**	
**Variables**	**r**	***p* value**	**r**	***p* value**	**r**	***p* value**
Aortic diameter at the subclavian artery	0.845	< 0.0001	0.824	< 0.0001	0.946	< 0.0001
Aortic diameter at the beginning of the aneursym	0.841	< 0.0001	0.854	< 0.0001	0.981	< 0.0001
Diameter of the thrombus within the aortic lesion	0.598	0.1118	0.992	< 0.0001	0.956	< 0.0001
Maximum diameter of the aneurysm	0.925	< 0.0001	0.895	< 0.0001	0.792	< 0.0001
Aortic diameter at the end of the aneurysm	0.729	< 0.0001	0.786	< 0.0001	0.971	< 0.0001
Aortic diameter at the coeliac trunk	0.720	< 0.0001	0.793	< 0.0001	0.989	< 0.0001
Diameter of the right femoral artery	0.426	0.0470	0.722	< 0.0001	0.836	< 0.0001
Diameter of the left femoral artery	0.395	0.0674	0.600	0.0005	0.404	0.0568
Distance of the aneurysm to the subclavian artery	0.544	< 0.0001	0.314	0.1216	0.987	< 0.0001
Length of the aneurysm	0.807	< 0.0001	0.954	< 0.0001	0.990	< 0.0001
Distance of the aneurysm to the coeliac trunk	0.805	< 0.0001	0.959	< 0.0001	0.989	< 0.0001
Total distance between the origin of the subclavian artery and the coeliac trunk	0.805	< 0.0001	0.892	< 0.0001	0.933	< 0.0001

Abbreviations: r, correlation coefficient.

In addition to the observations made at every single workstation, the repeatability of measurements between the three workstations was evaluated (Table 4 and Table 5). The 1st and 2nd rounds were considered separately from each other since this way a learning effect of the user could be shown that would have been lost in an all-encompassing comparison. Again, the measurement points “diameter of the right and left femoral artery” and “distance of the aneurysm to the subclavian artery” showed lower correlation coefficients than other measurement points. Furthermore, the high discrepancy between the 1st and 2nd round at the measurement points “maximum diameter of the aneurysm” and “diameter of the thrombus within the aortic lesion” is worth mentioning, showing better correlation coefficients in the 1st than the 2nd round. Direct comparisons revealed outstanding correlations for a few measurement points. However, these extraordinarily high correlation coefficients remained inconsistent, lacking a clear pattern between the two rounds.

**Table 4:** Correlation assessment per round comprising all three workstations, separated into the 1st and 2nd round.

**Variables**	**r (1st round)**	**r (2nd round)**
Aortic diameter at the subclavian artery	0.775	0.888
Aortic diameter at the beginning of the aneursym	0.789	0.900
Diameter of the thrombus within the aortic lesion	0.963	0.674
Maximum diameter of the aneurysm	0.854	0.521
Aortic diameter at the end of the aneurysm	0.856	0.719
Aortic diameter at the coeliac trunk	0.839	0.749
Diameter of the right femoral artery	0.509	0.410
Diameter of the left femoral artery	0.522	0.445
Distance of the aneurysm to the subclavian artery	0.519	0.564
Length of the aneurysm	0.891	0.912
Distance of the aneurysm to the coeliac trunk	0.856	0.983
Total distance between the origin of the subclavian artery and the coeliac trunk	0.810	0.912

Abbreviations: r, correlation coefficient.

**Table 5:** Pairwise correlation analysis of two workstations, respectively.

	**1st round**						**2nd round**					
	**General Electric vs. TeraRecon**		**General Electric vs. Siemens**		**TeraRecon vs. Siemens**		**General Electric vs. TeraRecon**		**General Electric vs. Siemens**		**TeraRecon vs. Siemens**	
	**r**	***p* value**	**r**	***p*****value**	**r**	***p* value**	**r**	***p* value**	**r**	***p* value**	**r**	***p* value**
Aortic diameter at the subclavian artery	0.710	< 0.0001	0.827	< 0.0001	0.795	< 0.0001	0.859	< 0.0001	0.868	< 0.0001	0.937	< 0.0001
Aortic diameter at the beginning of the aneursym	0.745	< 0.0001	0.784	< 0.0001	0.838	< 0.0001	0.912	< 0.0001	0.868	< 0.0001	0.923	< 0.0001
Diameter of the thrombus within the aortic lesion	0.966	< 0.0001	0.949	< 0.0001	0.973	< 0.0001	0.972	< 0.0001	0.615	0.0460	0.562	0.0573
Maximum diameter of the aneurysm	0.854	< 0.0001	0.798	< 0.0001	0.917	< 0.0001	0.919	< 0.0001	0.372	0.0186	0.415	0.0189
Aortic diameter at the end of the aneurysm	0.807	< 0.0001	0.579	0.0011	0.659	0.0003	0.646	0.0002	0.964	< 0.0001	0.521	0.0042
Aortic diameter at the coeliac trunk	0.732	< 0.0001	0.812	< 0.0001	0.954	< 0.0001	0.649	0.0002	0.951	< 0.0001	0.619	0.0002
Diameter of the right femoral artery	0.516	0.0461	0.507	0.0437	0.504	0.0445	0.305	0.0478	0.315	0.0069	0.614	0.0002
Diameter of the left femoral artery	0.718	< 0.0001	0.518	0.0412	0.338	0.1749	0.324	0.0212	0.346	0.0033	0.660	0.0003
Distance of the aneurysm to the subclavian artery	0.274	0.1028	0.887	< 0.0001	0.196	0.1505	0.819	< 0.0001	0.465	0.0017	0.538	0.0009
Length of the aneurysm	0.870	< 0.0001	0.876	< 0.0001	0.931	< 0.0001	0.947	< 0.0001	0.886	< 0.0001	0.905	< 0.0001
Distance of the aneurysm to the coeliac trunk	0.813	< 0.0001	0.794	< 0.0001	0.962	< 0.0001	0.982	< 0.0001	0.988	< 0.0001	0.977	< 0.0001
Total distance between the origin of the subclavian artery and the coeliac trunk	0.692	0.0002	0.863	< 0.0001	0.881	< 0.0001	0.907	< 0.0001	0.909	< 0.0001	0.918	< 0.0001

Abbreviations: r, correlation coefficient.

Combined processing times varied significantly between the two rounds. Comprising all workstations, the processing times of the 1st round were significantly longer than those of the 2nd round (237 vs. 142 min for General Electric, 189 vs. 156 min for TeraRecon, and 162 vs. 130 min for Siemens). In the 2nd round, processing times were found to be substantially reduced by 1 h and 35 min for General Electric (40% of the initial value, *p* < 0.0001), by 32 min for Siemens (20%, *p* = 0.0005), and by 33 min for TeraRecon (18%, *p* = 0.0008).

### 3.3 Error report

When looking for the sources of error, almost all system errors were reproducible on another workstation. Therefore, data sets may not have been compatible with the requirements of every single workstation. Since around half of the system errors occurred while the data set has been processed, a recurring error by the user has also to be considered.

A detailed error report is presented in Table 6.

**Table 6:** Error report comprising the need for manual correction of measurements as well as system errors.

	**General Electric**	**TeraRecon**	**Siemens**
**Need for manual correction**	**14**	**3**	**2**
Correction of the center axis	1	2	1
Correction of the captured vessel lumen	5	1	1
Other	8	0	0
			
**System errors**	**2**	**4**	**7**
While loading data sets	1	0	5
During image processing	1	4	1
Other	0	0	1

## 4 Discussion

This study systematically evaluated three different workstations regarding their diagnostic precision in-vivo and ex-vivo. Especially, the constructed phantom imitating the dimensions of a TAA facilitated the comparison of measurements with known fixed true values. In this context, measurements on the Siemens workstation deviated by 3.54% on average (*p* = 0.14) from the true size, those on TeraRecon by 4.86% (*p* < 0.0001), and on General Electric by 4.05% (or 6.01% including the detected user error at the measurement point “distance of the aneurysm to the artificial coeliac trunk”, *p* < 0.0001). Accordingly, Siemens was the most precise workstation regarding the deviation at simultaneously most fluctuating values (scattering of 4.46%). In comparison to the 1st round, processing times of the 2nd round were substantially reduced by 1 h and 35 min for General Electric (40% of the initial value, *p* < 0.0001), by 32 min for Siemens (20%, *p* = 0.0005), and by 33 min for TeraRecon (18%, *p* = 0.0008). Overall correlation coefficients between the 1st and 2nd round differed significantly (*p* = 0.037), reaching 0.898 for Siemens, 0.799 for TeraRecon, and 0.703 for General Electric.

### 4.1 Accuracy of the three different workstations

In addition to the precision of measurements, the calculated route of the central vessel axis is probably the most important technical criterion to provide reliable interventional planning. Since this step is largely automated, it requires extraordinary attention because the calculated central axis also serves as a simulation of catheter path positioning for the releasement of the stent over the aneurysm. For aneurysms and dissections that have a large and partially not clearly separable area to the true vascular lumen, the difficulty of correct delineation often arises in clinical practice. Attenuation differences between areas enhanced by contrast medium and surrounding tissue are frequently too weak for a clear differentiation by dedicated software algorithms resulting in suboptimal image quality. Additionally, if the contrast agent is not optimally recorded, it is also less likely that the software algorithms will make a correct assignment. In those cases, the overestimated vessel lumen distorts the calculated central axis of the vessel.

Both data records and the user himself are two influencing factors that promote or even cause errors. Using the phantom made it possible to minimize both of these sources of error which is one of the most important quality features of good image acquisition and postprocessing. Another factor that may have falsified our results is the rounding of all measurements on the Siemens workstation to whole millimeters, whereas measurements on the General Electric and the TeraRecon were carried out with a resolution of 0.1 mm. Therefore, the exclusion of fluctuations of less than a millimeter at the workstation from Siemens might have influenced the accuracy of measurements, particularly in the case of small vessels. As a consequence, the apparently precise limits of the scatter range can only be compared to a limited extent with those of the other workstations.

In a study about the accuracy of the TeraRecon workstation in patients with abdominal aortic aneurysms [14], the difference between pre-interventional planned stent length and true stent length was 4 mm on average (range, −1 to 23 mm). This corresponds to an average deviation of 2.5%, which is slightly better than ours. On the other hand, the scattering of values was more than twice as large as in our study. However, comparability to this study is limited since the authors used a different study design without an additional phantom. All comparisons were not related to the true size but to a new CT dataset of patients after successful endograft implantation.

The main advantage of the phantom used in our study was the knowledge about its known and verifiable dimensions, which allowed for a direct comparison of the acquired individual measurements from each workstation with the true dimensions of the phantom.

### 4.2 Handling and user experience

Considering the complete lack of user experience in the 1st round, the course of time savings in measurements hereafter suggest that the software solutions of the workstations are much more difficult to use for untrained investigators without previous in-depth training.

Furthermore, our practical experience shows that there is a clear reluctance to use special applications and functions of these workstations as long as the users have not undergone specific training. Additionally, physicians are often under a certain time pressure in their clinical routine avoiding too long processing times on a workstation that is new to them. Therefore, the accuracy and precision of measurements on different workstations for one patient are rarely compared by a single user. The motivation for our study resulted from this lack of data and experience.

Regarding the user-friendliness of the individual software solutions, listing bug fixes during image evaluation is just a limited attempt to put this part of our examinations into a sober framework. With the possibility of manual corrections, the question arose to what extent work processes should be considered normal or corrective within an examination. In this context, automatization levels of the working process vary at the different workstations because working steps that always occur as a fixed component at one workstation would already be seen as manual corrections to the workflow at another. It could be argued that due to the nature and conceptualization of the software, manual corrections at the workstation from General Electric are an integral part of the regular workflow. Exactly the opposite was the case with the Siemens workstation. Due to the software design, most of the measurements were taken by the investigator himself. Therefore, a correction was unnecessary in most cases since the user already did the first measurement by himself.

### 4.3 Transfer into clinical practice

Our study has several important clinical implications. In addition to the precision of measured values, the direction in which the measurement error deviates from the true value also plays an important role in the clinical routine. Positive deviations in the diameters are better than negative ones to a certain extent of approximately 10–15% [15]. A stent with a too-large diameter in this range leads to a higher contact pressure on the vessel wall and thus to more stability. On the other hand, stent diameters that are too small can lead to inadequate anchoring of the endoprosthesis and finally to dislocation [16].

In the case of length measurements, the situation is reversed. Stents that are too long would increase the risk of vessel obstruction proximal or distal to the insertion zone. On the contrary, a stent that is too short may not be long enough to cover the pathology. In our study, it has been shown very clearly that the diameters were usually determined too large and the distances too short on all workstations. In this context, a workstation deviation that is relatively constant can be used in practice, bearing in mind that the results must be corrected for the value of the deviation. A deviation that is accompanied by inconstant fluctuations is far more difficult to use because this error cannot be consistently corrected.

### 4.4 Limitations

Several limitations have to be addressed when interpreting our results. First, the experiments were carried out by a single experienced radiologist who was familiar with the handling of workstations. Therefore, the results might not be transferable to investigations conducted by inexperienced users. Second, the repetition of examinations at every single workstation has led to a continuously growing training effect of potentially difficult patient data sets and recall bias. Due to the similarities between the software, learning effects are also transferred from one workstation to the next. Therefore, our results require randomized prospective validation including a larger number of patients for a more in-depth analysis. Third, despite careful measurement of diameters in positions without air bubbles, residual errors cannot be fully excluded. Finally, the investigated study population was relatively small. Future studies are required to validate our preliminary study results.

## 5 Conclusions

In summary, all three workstations facilitated accurate distance determinations in the majority of cases at simultaneously high reproducibility. However, Siemens provided the most precise workstation with the lowest deviation from true vessel dimensions and the highest correlation between the 1st and 2nd round of measurements. Therefore, pre-interventional planning of TEVAR in patients with TAAs using three-dimensional CTA is feasible and can obviate the need for invasive aortography.

## CRediT authorship contribution statement

**VK**: Writing - Original Draft, Supervision, Project administration, Software. **GL** and **JES**: Data Curation, Investigation. **LDG**: Visualization, Resources. **KE, TDA, CB, SB, SM, SSM, MH**, and **MHA**: Formal analysis, Methodology, Software, Validation. **SZ, AT, IY**, and **JES**: Project administration, Validation. **TJV** and **TGR**: Writing - Review & Editing, Methodology, and Conceptualization.

## Ethical statement

The present work has been carried out in accordance with The Code of Ethics of the World Medical Association (Declaration of Helsinki) and is in line with the Recommendations for the Conduct, Reporting, Editing and Publication of Scholarly Work in Medical Journals. Moreover, the work aims for the inclusion of representative human populations (sex, age, and ethnicity) as per those recommendations. The terms sex and gender are used correctly.

The institutional ethical review board approved this retrospective study. The need for written informed consent was waived. The privacy rights of human subjects have been always observed.

## Funding Statement

No funding has been received for this project.

## Appendix A Supplementary material

.

Figure S1 Exemplary full-screen view of a postprocessing step at the TeraRecon workstation. A coronal and sagittal cross-section of the aorta with a calculated central axis can be seen on the left side of the image. Two cross-sectional areas of the selected vessels are shown below (bottom left). Centered at the top, the central axis is projected into a three-dimensional reconstruction with fixed points for the measurements set below. The panels on the right show the selection of the template and the individual working steps. Supplementary material

.

Figure S2 Screen view during image processing on the Siemens workstation. In the two panels on the left side of the image, axial cross-sections along the central axis are shown. In the right panels, the corresponding central axis and adjacent structures around the vessel are depicted. Different display options and the "Vessel Analysis" menu for the measurements are illustrated on the right side of the screen. Supplementary material

.

Figure S3 Images recorded during the processing of the datasets at the workstation from General Electric. A) Three-dimensional reconstruction of the aorta after removing adjacent structures using adjusted Hounsfield unit settings. B) Coronal section. C) A deformed, "stretched” view of the aorta. In addition, the orthogonal planes to the central axis are shown here, on which the diameters and the endpoints of the distances are located. Supplementary material
