# Supplementary material for: Comprehensive comparison of three different workstations for accurate planning of endovascular stent implantation in patients with thoracic aortic aneurysms
Source: Eur J Radiol Open. 2022 Jun 16;9:100427. doi: 10.1016/j.ejro.2022.100427 (PMC9213697; doi:10.1016/j.ejro.2022.100427)
Supplement: Supplementary file 1 — Supplementary material [file mmc1.docx]

***Supplemental material***

1. **Aquarius (Version 3.7.0.12; TeraRecon, Durham, USA)**

The Aquarius software provides a template for three-dimensional aortic analysis. When this template is applied to a CT data set, the software first creates a three-dimensional projection of the spiral CT data. The missing gaps between the recorded layers are interpolated by the software from data of the adjacent structures. If the gaps between the layers become too large, the calculated picture becomes unrealistic and is no longer suitable for serious planning of interventions. In such a case, the software warns the user against using this CT data set for further processing.

If the “aneurysm” protocol is selected by the user, the software removes those portions of the three-dimensional image which, based on their Hounsfield units, are unlikely to correspond to the contrast-enhanced aorta. The final result depends heavily on the incoming data set, but the range of Hounsfield units can be individually adjusted. This option enables the selection of the aorta and its vascular tree as precisely as possible.

As a next step, the investigator can use the three-dimensional image or two-dimensional sectional images of the aorta to establish fixed points within the aortic lumen. The software calculates the central axis of the vessel according to the set of fixed points. In cases of pronounced deformations or artifacts, the user himself has to insert further fixed points along a realistic central axis. Therefore, it is essential to track the measurements across the entire aorta in its cross-sectional images to check all automated working steps and make manual corrections if necessary.

In addition to the three-dimensional and cross-sectional visualization, the workstation offers the option of a “stretched view” which represents a stretched sagittal view of the aorta along the central axis ***(Supplemental Figure S1)***. The deformation of the curved central axis of the aorta into a straight line results in a distortion of the image. However, it simplifies the overview and the length measurements of the aorta enormously, so that the distortion of the vessel wall is accepted for this working step.

In the next step, ready-made measurement protocols can be selected to make the sequence of steps in measuring the aorta easier. Vessel diameters are measured simply by selecting a position on the central axis of the vessel. Distances along the aorta, on the other hand, must be determined manually by setting start- and endpoints. It is up to the user to decide which measurements can be taken, as the protocols only recommend the sequence. If all measurements have been taken satisfactorily, a measurement log file can be saved and printed. In addition, images of the three-dimensional model and all measurements are saved. As part of the measurement protocols, TeraRecon cooperates with leading manufacturers of endovascular aortic stents. Appropriate measurement protocols are available for individual products. The measurement protocol “Medtronic Vailant TAA” was used in this work.

1. **Syngo (Version VE32B; Siemens Healthineers, Forchheim, Germany)**

Like the competing models, this workstation from Siemens offers a plethora of applications and functions. The function for analyzing the aorta is located in the “InSpace 4dct” sub-program. Templates for the visualization of the aorta can be selected within the InSpace program. By choosing the "vessel analysis" function one reaches the basic software module for the assessment of the aorta. For a selective display, limit values ​​for Hounsfield units can be specified here. The user is not dependent on finding a usable three-dimensional image but can also work in the conventional two-dimensional images in all planes. The next step is the determination of the central axis. What differs from the other workstations is that the calculated central axis is not displayed in a "stretched view". The measurements are all made on the original three- and two-dimensional images of the aorta. Diameters are calculated by the software simply by setting a point on the central axis. If the software does not succeed in sufficiently recognizing the cross-sectional area of ​​the vessel, the user can adjust it by moving boundary points.

In contrast to the other workstations, no protocol templates are available for the measurements. The user decides for himself how many measurements he takes in any order. An illustration of the user interface is depicted in ***Supplemental Figure S2***.

Measurement results and recordings of the processed images are also stored within the workstation and can be exported to the PACS system (PACS Centricity, Version 4.2; General Electric Healthcare, Solingen, Germany).

1. **Volume Share 2 (Version AW.4.4; GE Healthcare, Waukesha, USA)**

The workstation Volume Share 2 is similar in its logical and graphical structure to the Aquarius workstation from TeraRecon. The sub-program "Volume Viewer" opens with a wide choice of display options that have specifically been prepared for the individual processing of different anatomical regions, including the option “aortic analysis before stent”. The selected data set is automatically reduced to the aorta and its larger branches in the three-dimensional image. This representation is accompanied by two-dimensional images in sagittal, coronal, and transversal planes. Based on the different gray levels of contrast-enhanced tissues and the surrounding structures, the data set is reduced to the Hounsfield unit area, which usually corresponds to that of the contrast-enhanced vessel.

The software also supports the semiautomatic measurement of distances and diameters. In practice, this means that the start- and endpoints of the routes are set by the user and the distance is calculated based on anatomical relationships. To measure the diameters of aortic cross-sections, the user is only asked to specify the individual position along the central axis.

In addition, this workstation offers the possibility of placing several fixed points in the large aortic branches. From this, the software calculates a network of central axes, so that the large outgoing vessels can be included in the planning process. This is particularly useful in the case of pathologies that penetrate these vessels.

After determining the positions of the diameters to be measured along the central axis, the distances between the individual diameters are also calculated immediately. Since the required distance measurements often correspond to the distances between the diameter positions, this automation usually saves one working step. Volume Share 2 also has the "stretched view" function ***(Supplemental Figure S3)***.

After finishing measurements, a report of the results is created in DICOM format, which, in addition to the numerical values, contains images on which each measurement can be graphically reproduced. The image series of the protocol can be sent to the PACS system and viewed like conventional CT data sets from clinical routine.
