# Supplementary material for: Comprehensive comparison of three different workstations for accurate planning of endovascular stent implantation in patients with thoracic aortic aneurysms
Source: Eur J Radiol Open. 2022 Jun 16;9:100427. doi: 10.1016/j.ejro.2022.100427 (PMC9213697; doi:10.1016/j.ejro.2022.100427)
Supplement: Supplementary file 2 [file mmc2.docx]

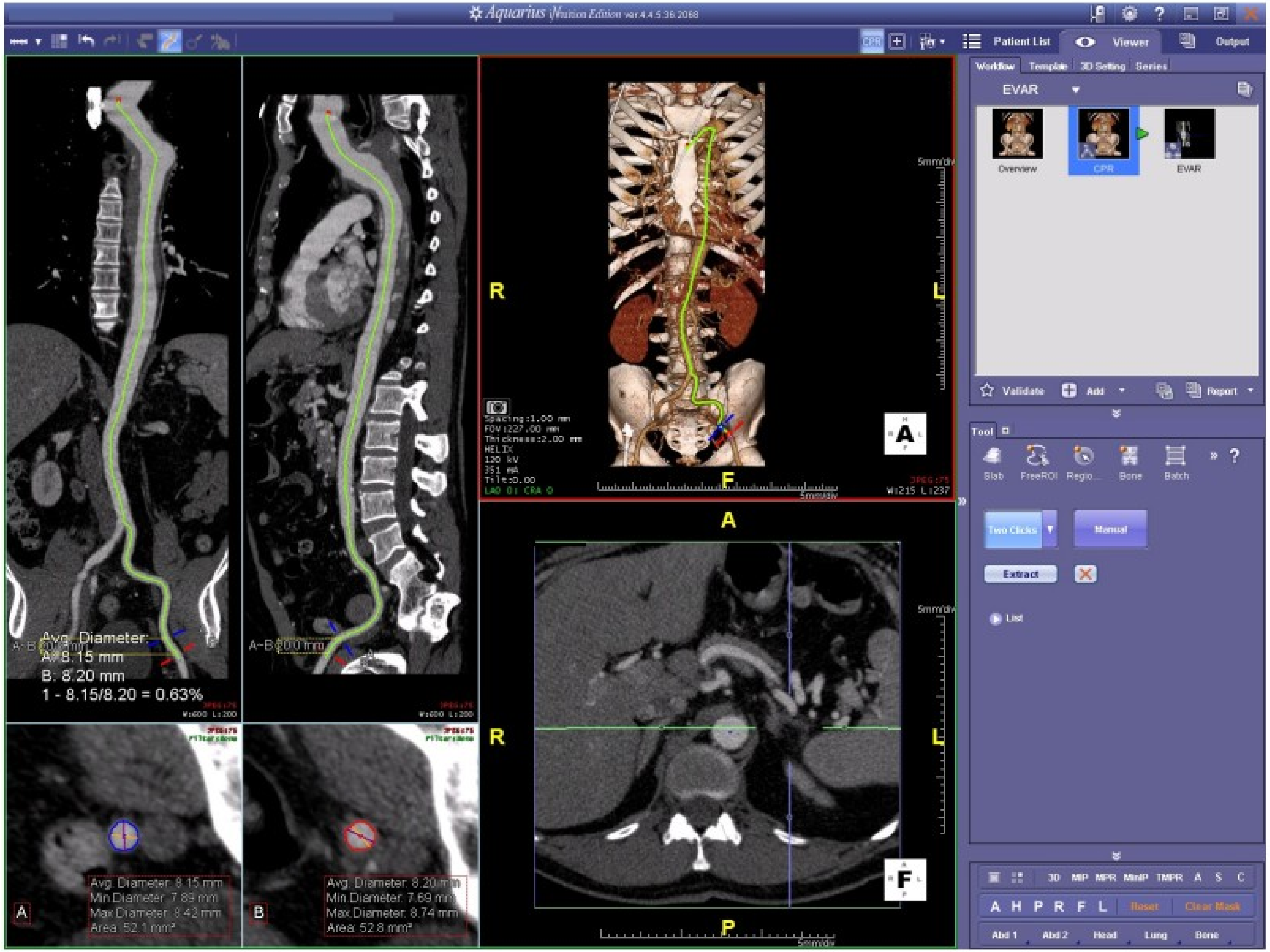
Patient List
Viewer
Output
Workflow
Template
3D Setting
Series
EVAR
Overview
CPR
EVAR
Validate
Add
Report
Tool
Slab
FreeROI
Bone
Batch
Two Clicks
Manual
Extract
List
R
L
F
A
P
Avg. Diameter:
A: 8.15 mm
B: 8.20 mm
1 - 8.15/8.20 = 0.63%
Avg. Diameter: 8.15 mm
Min Diameter: 7.89 mm
Max Diameter: 8.42 mm
Area: 52.1 mm²
Avg. Diameter: 8.20 mm
Min Diameter: 7.69 mm
Max Diameter: 8.74 mm
Area: 52.8 mm²
3D MIP MPR MinIP TMPR A S C
A H P R F L Reset Clear Mask
Abd 1 Abd 2 Head Lung Bone
