# Supplementary material for: Comprehensive comparison of three different workstations for accurate planning of endovascular stent implantation in patients with thoracic aortic aneurysms
Source: Eur J Radiol Open. 2022 Jun 16;9:100427. doi: 10.1016/j.ejro.2022.100427 (PMC9213697; doi:10.1016/j.ejro.2022.100427)
Supplement: Supplementary file 3 [file mmc3.docx]

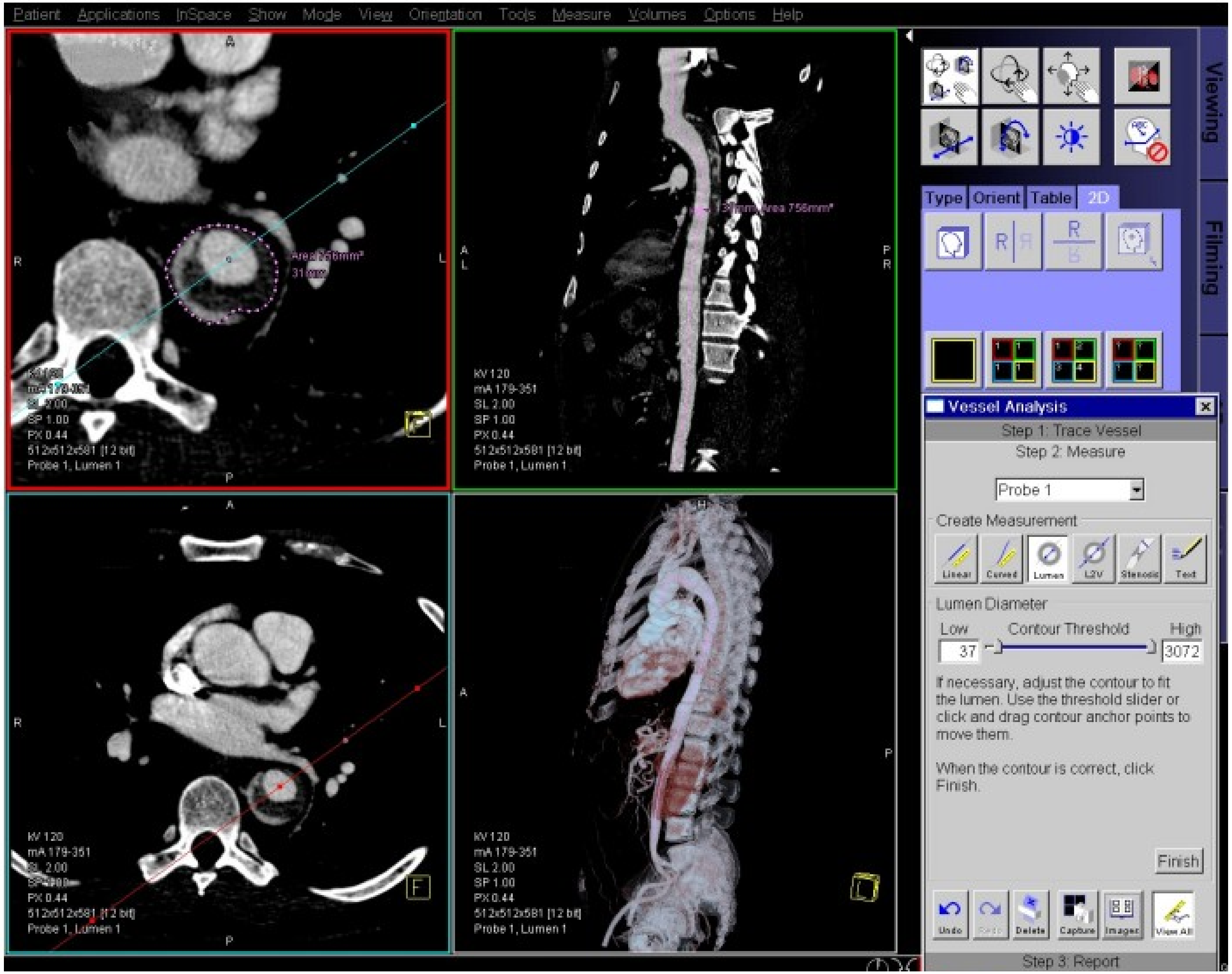
Patient Applications InSpace Show Mode View Orientation Tools Measure Volumes Options Help
Viewing
Filming
Type Orient Table 2D
kV 120
mA 179-351
SL 2.00
SP 1.00
PX 0.44
512x512x581 [12 bit]
Probe 1, Lumen 1
Vessel Analysis
Step 1: Trace Vessel
Step 2: Measure
Probe 1
Create Measurement
Linear
Curved
Lumen
L2V
Stenosis
Text
Lumen Diameter
Low
Contour Threshold
High
37
3072
If necessary, adjust the contour to fit the lumen. Use the threshold slider or click and drag contour anchor points to move them.
When the contour is correct, click Finish.
Finish
Undo
Redo
Delete
Capture
Images
View All
Step 3: Report
