# Supplementary material for: Comprehensive comparison of three different workstations for accurate planning of endovascular stent implantation in patients with thoracic aortic aneurysms
Source: Eur J Radiol Open. 2022 Jun 16;9:100427. doi: 10.1016/j.ejro.2022.100427 (PMC9213697; doi:10.1016/j.ejro.2022.100427)
Supplement: Supplementary file 4 [file mmc4.docx]

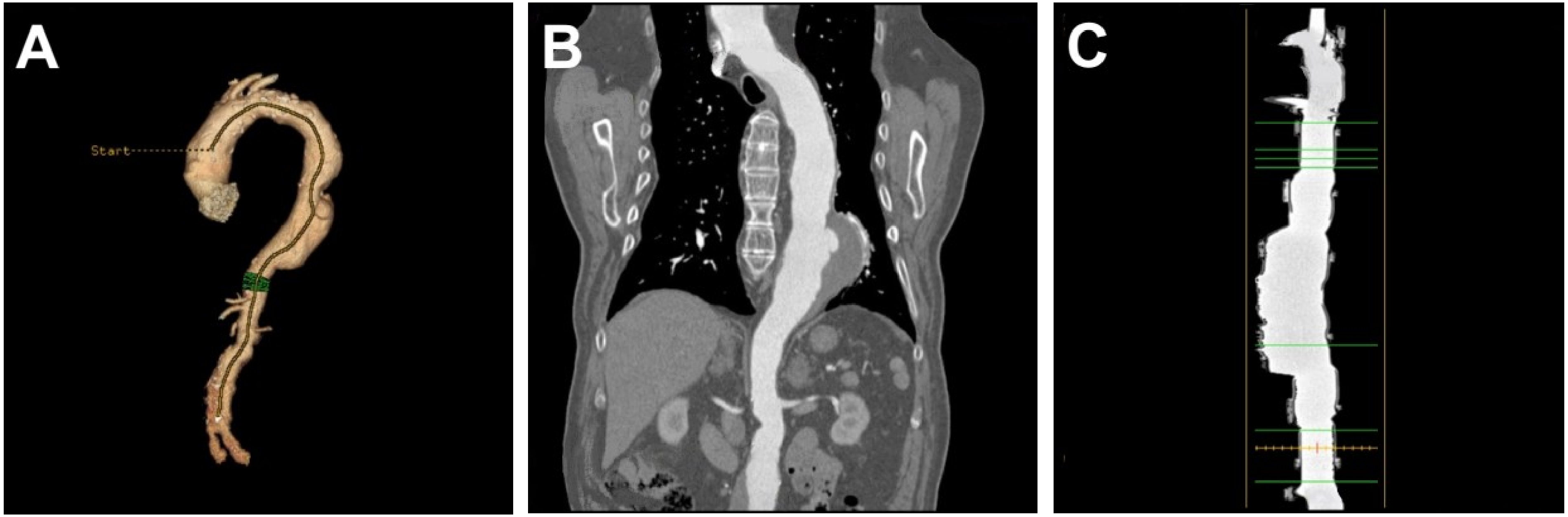
A
Start
B
C
